# Identification and Validation of Novel Cerebrospinal Fluid Biomarkers for Staging Early Alzheimer's Disease

**DOI:** 10.1371/journal.pone.0016032

**Published:** 2011-01-12

**Authors:** Richard J. Perrin, Rebecca Craig-Schapiro, James P. Malone, Aarti R. Shah, Petra Gilmore, Alan E. Davis, Catherine M. Roe, Elaine R. Peskind, Ge Li, Douglas R. Galasko, Christopher M. Clark, Joseph F. Quinn, Jeffrey A. Kaye, John C. Morris, David M. Holtzman, R. Reid Townsend, Anne M. Fagan

**Affiliations:** 1 Division of Neuropathology, Washington University School of Medicine, St. Louis, Missouri, United States of America; 2 Department of Pathology and Immunology, Washington University School of Medicine, St. Louis, Missouri, United States of America; 3 Knight Alzheimer's Disease Research Center, Washington University School of Medicine, St. Louis, Missouri, United States of America; 4 Department of Neurology, Washington University School of Medicine, St. Louis, Missouri, United States of America; 5 Department of Medicine, Washington University School of Medicine, St. Louis, Missouri, United States of America; 6 Division of Metabolism and Proteomics Center, Washington University School of Medicine, St. Louis, Missouri, United States of America; 7 Hope Center for Neurological Disorders, Washington University School of Medicine, St. Louis, Missouri, United States of America; 8 Department of Veterans Affairs Northwest Network Mental Illness Research, Education, and Clinical Center, University of Washington School of Medicine, Seattle, Washington, United States of America; 9 Department of Psychiatry and Behavioral Sciences, University of Washington School of Medicine, Seattle, Washington, United States of America; 10 Department of Neurosciences, University of California at San Diego, San Diego, California, United States of America; 11 Department of Neurology, University of Pennsylvania, Philadelphia, Pennsylvania, United States of America; 12 Alzheimer's Disease Center, University of Pennsylvania, Philadelphia, Pennsylvania, United States of America; 13 Avid Radiopharmaceuticals, Philadelphia, Pennsylvania, United States of America; 14 Layton Aging and Alzheimer's Disease Center, Oregon Health Science University, Portland, Oregon, United States of America; National Institute on Aging Intramural Research Program, United States of America

## Abstract

**Background:**

Ideally, disease modifying therapies for Alzheimer disease (AD) will be applied during the ‘preclinical’ stage (pathology present with cognition intact) before severe neuronal damage occurs, or upon recognizing very mild cognitive impairment. Developing and judiciously administering such therapies will require biomarker panels to identify early AD pathology, classify disease stage, monitor pathological progression, and predict cognitive decline. To discover such biomarkers, we measured AD-associated changes in the cerebrospinal fluid (CSF) proteome.

**Methods and Findings:**

CSF samples from individuals with mild AD (Clinical Dementia Rating [CDR] 1) (n = 24) and cognitively normal controls (CDR 0) (n = 24) were subjected to two-dimensional difference-in-gel electrophoresis. Within 119 differentially-abundant gel features, mass spectrometry (LC-MS/MS) identified 47 proteins. For validation, eleven proteins were re-evaluated by enzyme-linked immunosorbent assays (ELISA). Six of these assays (NrCAM, YKL-40, chromogranin A, carnosinase I, transthyretin, cystatin C) distinguished CDR 1 and CDR 0 groups and were subsequently applied (with tau, p-tau181 and Aβ42 ELISAs) to a larger independent cohort (n = 292) that included individuals with very mild dementia (CDR 0.5). Receiver-operating characteristic curve analyses using stepwise logistic regression yielded optimal biomarker combinations to distinguish CDR 0 from CDR>0 (tau, YKL-40, NrCAM) and CDR 1 from CDR<1 (tau, chromogranin A, carnosinase I) with areas under the curve of 0.90 (0.85–0.94 95% confidence interval [CI]) and 0.88 (0.81–0.94 CI), respectively.

**Conclusions:**

Four novel CSF biomarkers for AD (NrCAM, YKL-40, chromogranin A, carnosinase I) can improve the diagnostic accuracy of Aβ42 and tau. Together, these six markers describe six clinicopathological stages from cognitive normalcy to mild dementia, including stages defined by increased risk of cognitive decline. Such a panel might improve clinical trial efficiency by guiding subject enrollment and monitoring disease progression. Further studies will be required to validate this panel and evaluate its potential for distinguishing AD from other dementing conditions.

## Introduction

Clinicopathological studies suggest that Alzheimer's disease (AD) pathology (amyloid plaque formation, followed by gliosis and neurofibrillary tangle formation) begins 10–15 years before the onset of very mild dementia [1], [2]. This period of ‘preclinical AD’ could provide an opportunity for disease modifying therapies to prevent or forestall the synaptic and neuronal losses associated with cognitive impairment [3]–[5]. However, before such interventions can be developed and judiciously administered, accurate tools must be in place to diagnose and monitor the pathophysiological condition of individuals with preclinical AD and very early stage AD dementia. Clinical examination cannot detect preclinical disease or measure cellular and molecular changes within the brain, and, in general, has limited accuracy when diagnosing the very earliest symptomatic stages of AD. Therefore, there is an urgent need to identify biomarkers that can do so. Because its composition is rapidly and directly influenced by the brain, the cerebrospinal fluid (CSF) proteome represents an appealing source for such biomarkers.

Indeed, a few CSF proteins have already shown promise as diagnostic biomarkers for clinical AD (dementia of the Alzheimer type [DAT]) and even preclinical AD. Lower mean levels of CSF Aβ42 and higher mean levels of tau and phosphorylated tau can distinguish groups with DAT from cognitively normal controls [6], [7]. Unfortunately, value ranges for each biomarker show substantial overlap between groups.

Recently, using positron-emission tomography PET imaging with Pittsburgh Compound B (PIB) to measure brain amyloid in vivo, we and others have demonstrated that low CSF Aβ42 can serve as an indicator of amyloid deposition [8]–[13], and that CSF tau levels correlate positively with in vivo brain amyloid load [11], [14]. Importantly, both of these associations are independent of clinical diagnosis [8]–[11], though CSF tau does correlate with more sensitive measures of cognition [14]. These findings suggest that the overlap of biomarker values between clinical groups may, in part, reflect “contamination” of control groups by cognitively normal individuals exhibiting amyloid plaques and early neurodegeneration (preclinical AD), low CSF Aβ42 and elevated CSF tau. Supporting this notion, elevated ratios of tau/Aβ42 and p-tau181/Aβ42 (consistent with the presence of amyloid plaques and neurodegeneration) have been associated with increased risk of converting from cognitive normalcy to mild cognitive impairment or dementia [9], [15], and with increased rate of cognitive decline among those with very mild dementia [16]. Together, these findings suggest that CSF biomarkers can describe neuropathological state and trajectory. They also suggest that a pathological staging system based on biomarkers might be a favorable alternative or adjunct to clinical staging for guiding treatment decisions or designing clinical trials.

Beyond amyloid plaque formation, other features of AD pathophysiology might also be exploited as therapeutic targets, sources of diagnostic biomarkers, or measures of disease progression. In addition to Aβ42 and tau, many other candidate AD biomarkers have been identified by either targeted or unbiased proteomics screens [17]–[27]. Only a few of these studies have tested large, well-characterized cohorts, however. Even fewer have evaluated biomarkers for their ability to distinguish the very early stages of AD pathophysiology. Thus, there remains a critical need for validated AD biomarkers that can properly categorize individuals by early pathological stage; such markers may have potential for monitoring neuropathological decline and, thereby, for evaluating response to disease-modifying therapies.

The goal of this study, therefore, is to identify such CSF protein biomarkers for AD using the unbiased proteomic technique of two-dimensional difference-in-gel electrophoresis (2D-DIGE) coupled with liquid chromatography and tandem mass spectrometry (LC-MS/MS), and to evaluate them further in a larger independent cohort using quantitative enzyme-linked immunosorbent assays (ELISA). Our findings suggest that a small ensemble of novel biomarkers may be able to distinguish several stages of cognitive decline in early AD, and improve the ability of current leading biomarkers tau and Aβ42 to discriminate early symptomatic AD from cognitive normalcy.

## Methods

### Ethics Statement

The study protocols were approved by the institutional review boards of the University of Washington, the Oregon Health and Science University, the University of Pennsylvania, the University of California San Diego, and Washington University. Written informed consent was obtained from all participants at enrollment. All aspects of this study were conducted according to the principles expressed in the Declaration of Helsinki.

### Participant Selection for Discovery Cohort

Participants (n = 48), community-dwelling volunteers from University of Washington [n = 18], Oregon Health and Science University [n = 11], University of Pennsylvania [n = 11], and University of California San Diego [n = 8], were 51–87 years of age and in good general health, having no other neurological, psychiatric, or major medical diagnoses that could contribute to dementia, nor use of exclusionary medications (e.g. anticoagulants) within 1–3 months of lumbar puncture (LP). Cognitive status was evaluated based on criteria from the National Institute of Neurological and Communicative Diseases and Stroke-Alzheimer's Disease and Related Disorders Association [28]. In the morning after overnight fasting, CSF was obtained by LP, collected and aliquoted in polypropylene tubes, and immediately frozen at −80°C. Participants who were cognitively normal (Clinical Dementia Rating [CDR] of 0 [n = 24]) [29], or had mild “probable AD” (CDR 1) (n = 24), were selected from a larger group of 120 individuals on the basis of CSF Aβ42 (relatively high and low values, respectively), and, when possible, CSF tau (relatively low and high values, respectively) to increase the likelihood of CDR 1 participants having and CDR 0 participants not having AD pathology. CSF Aβ42 and tau levels for the discovery cohort were all measured in a single laboratory using well-established ELISA assays ([30] and Innotest, Innogenetics, Ghent, Belgium). Although quantitative thresholds were not defined prior to sample selection, the lowest CDR 0 value and the highest CDR 1 value for CSF Aβ42 in this ‘discovery cohort’ were 609 and 361 pg/mL, respectively; ranges for CSF tau were 141–461 pg/mL for CDR 0 and 215–1965 pg/mL for CDR 1.

### Participant Selection for Validation Cohort

Participants (n = 292), community-dwelling volunteers enrolled at the Knight Alzheimer Disease Research Center at Washington University (WU-ADRC), were ≥60 years of age and met the same exclusion criteria as the discovery cohort. The study protocol was approved by the Human Studies Committee at Washington University, and written and verbal informed consent was obtained from participants at enrollment. Cognitive status was determined as with the discovery cohort. Participants who were cognitively normal (CDR 0, n = 198), very mildly demented (CDR 0.5, n = 65) or mildly demented (CDR 1, n = 29) at the time of LP were selected without regard to previously measured biomarkers. Some CDR 0.5 participants met criteria for mild cognitive impairment (MCI) and some showed even milder impairment, and could be considered “pre-MCI” [31]. All CDR 1 individuals had received a diagnosis of DAT (See Table 1 for demographic characteristics). Apolipoprotein E (*APOE*) genotypes were determined by the WU-ADRC Genetics Core. Fasted CSF (20–30 mL) was collected, gently mixed, centrifuged, aliquoted and frozen at −80°C in polypropylene tubes [9].

**Table 1:** Demographic, clinical, genotype characteristics of validation cohort.

Characteristic	CDR 0	CDR 0.5	CDR 1
Number of Participants	198	65	29
Gender (% Female)	63%	54%	52%
*APOE* genotype, % ε4 positive	35%	51%	59%
Mean MMSE score (SD)	28.9 (1.3)	26.3 (2.8)	22.3 (3.9)
Mean age at LP (SD), years	71.0 (7.3)	73.8 (6.8)	76.5 (6.2)
Mean CSF Aβ42 (SD), pg/mL	605 (240)	446 (230)	351 (118)
Mean CSF tau (SD), pg/mL	304 (161)	539 (276)	552 (263)
Mean CSF p-tau181 (SD), pg/mL	55 (25)	85 (44)	77 (38)

**Abbreviations:** CDR, Clinical Dementia Rating; CDR 0, cognitively normal; CDR 0.5, very mild dementia; CDR 1 mild dementia; APOE, apolipoprotein E; MMSE, Mini-Mental State Examination; LP, lumbar puncture; SD, standard deviation; CSF, cerebrospinal fluid; Aβ42, amyloid beta 42 peptide; p-tau181, tau phosphorylated at threonine 181.

### Multi-Affinity Immunodepletion of CSF

A pooled CSF sample, containing an equivalent volume from every ‘discovery’ cohort sample, was prepared as an internal standard for 2D-DIGE to facilitate the matching of gel features, and to allow normalization of the intensity of each gel feature among different gels. To enrich for proteins of low-abundance prior to 2D-DIGE, each CSF sample was depleted of six highly-abundant proteins (albumin, IgG, IgA, haptoglobin, transferrin, and α-1-antitrypsin) by immunoaffinity chromatography (Agilent Technologies, Palo Alto, CA) according to the manufacturer's instructions and as described previously [32]. Depleted samples were then concentrated using 10 kDa exclusion filters to retain larger molecules. As a ‘benchmark’ of immunodepletion column performance, an aliquot of reference CSF was depleted after every group of seven experimental chromatographic depletions. Non-depleted reference CSF, depleted CSF and the proteins that were retained by the column were analyzed by 2D-DIGE as previously described [32], [33]; gel images obtained from all reference CSF depletion analyses were similar (data not shown), indicating consistent column performance over time.

### 2D-DIGE

2D-DIGE was performed as described previously [32], [33]. Briefly, CDR 0 and CDR 1 samples were randomly paired. 50 micrograms of protein from each paired sample and from an aliquot of the pooled CSF sample were labeled with one of three N-hydroxysuccinimide cyanine dyes. The labeled proteins and 100 micrograms of unlabeled protein from each sample were mixed and equilibrated with an immobilized pH gradient strip for isoelectric focusing (first dimension), after which the strip was treated with reducing and alkylating solutions prior to SDS-PAGE (second dimension). Cy2, Cy3 and Cy5-labeled images were acquired on a Typhoon 9400 scanner (GE Healthcare, United Kingdom) at excitation/emission wavelengths of 488/520, 532/580, and 633/670 nm, respectively.

### Gel Image and Statistical Analysis

The comparative two-dimensional gel analysis was performed using an established experimental design [34] in which the high variation between gels is minimized by including a common, labeled pooled sample in all gels. Intra-gel feature detection, quantification and inter-gel matching and quantification were performed using the Differential In-Gel Analysis (DIA) and Biological Variation Analysis (BVA) modules of DeCyder software v 6.5 (GE Healthcare), respectively, as described previously [32]. This process (DIA analysis) resulted in approximately 5,000 gel features per gel image. In five gels, one sample contained significant amounts of hemoglobin indicating possible blood contamination. Therefore, all images from gels with these hemoglobin-containing samples were removed from further analysis. Remaining gel images were separated into three sets: standard (pool of all samples), CDR 0 and CDR 1. The pooled sample image with the largest number of well-resolved gel features was chosen as a master image. Gel features in each remaining pooled sample image were hand matched to gel features in the master image. For each gel feature that was matched across >50% of the gels (n = 764), a Student's t-test (α = 0.05) was performed to determine the statistical significance of CDR 0/CDR 1 ratios, using the DeCyder EDA (Extended Data Analysis) module. To maximize discovery rate and minimize type II error, no multiple test correction was applied. The image intensity data for the statistically significant gel features (n = 119) were then subjected to unsupervised hierarchical clustering (DeCyder EDA module).

### Protein/Peptide Identification by LC-MS/MS

Gel features with significant intensity differences were targeted by a robotic gel sampling system (ProPic; Genomics Solutions, Ann Arbor, MI) and transferred into 96 well plates for in-gel digestion with trypsin using a modification of a method [35] described previously [33]. Aliquots of these digests were processed for and analyzed by LC-MS/MS using a capillary LC (Eksigent, Livermore CA) interfaced to a nano-LC-linear quadrupole ion trap Fourier transform ion cyclotron resonance mass spectrometer (nano-LC-FTMS) [36] QStar [37] or LTQ [36]. The tandem spectra were searched against the National Center for Biotechnology Information non-redundant protein database NR (downloaded on 02-18-2007) using MASCOT, version 2.2.04 (Matrix Sciences, London). The database searches were constrained by allowing for trypsin cleavage (with up to two missed cleavage sites), fixed modifications (carbamidomethylation of Cys residues) and variable modifications (oxidation of Met residues and N-terminal pyroglutamate formation). Protein identifications were considered genuine if at least two peptides were matched with individual MASCOT ion scores ≥40.

Using nano-LC-MS/MS, multiple proteins were identified in the majority of individual gel features. The frequent observation of multiple proteins in single gel features was attributed to the sensitivity and greater peptide coverage that can be achieved with nano-LC-MS methods as compared to, for example, MALDI-MS analysis of peptides from gel features. Assignment of the major protein(s) from each gel feature was achieved using quantitative proteomics from spectra counting [38]. The detection of multiple proteins within single gel features could also be attributed to artifacts and technical issues associated with 2D gel electrophoresis: 1) incomplete resolution of proteins by gel electrophoresis (due to similar charge and size characteristics, excessive abundance of neighboring proteins, or artifactual streaking); 2) changes in molecular weight associated with cyanine dye labeling, particularly for lower molecular weight proteins; and 3) sample ‘carryover’ during robotic gel sampling or during nano-LC-MS/MS.

All relevant proteomics data are detailed in Table S1.

### Enzyme Linked Immunosorbent Assays (ELISAs) and Statistical Analyses

CSF samples were analyzed by ELISA in duplicate for Aβ42, total tau, and phospho-tau181 (Innotest, Innogenetics, Ghent, Belgium) after one freeze-thaw cycle, and in triplicate for all other ELISAs after two freeze-thaw cycles. Samples were evaluated using commercially available ELISAs for NrCAM (R&D Systems Inc., Minneapolis, MN), YKL-40 (Quidel Corporation, San Diego, CA), apolipoprotein E (Medical and Biological Laboratories Company, Ltd., Nagoya, Japan), clusterin/apolipoprotein J (ALPCO Diagnostics, Salem, NH), pigment epithelium-derived factor (PEDF)/serpin-F1 (Chemicon International Inc./ Millipore Corporation, Billerica, MA), beta-2 microglobulin (ALPCO Diagnostics), ceruloplasmin (Assaypro, St. Charles, MO), chromogranin A (ALPCO Diagnostics, low binding capacity manufacturing protocol), transthyretin (Assaypro), and cystatin C (US Biological, Swampscott, MA), according to manufacturer's instructions, with adjustments for the analysis of CSF. A sandwich ELISA was developed for carnosinase I using goat anti-human carnosinase I antibody (2 µg/mL, R&D Systems Inc.) for capture, rabbit anti-human carnosinase I antibody (1 µg/mL, Sigma-Aldrich Corporation, St. Louis, MO) for detection, goat anti-rabbit:horseradish peroxidase (1∶5000, Upstate Biologicals Inc./Millipore Corporation) for reporting, and TMB (3,3′,5,5′-tetramethylbenzidine) Super Slow (Sigma-Aldrich Corporation) for color development; recombinant carnosinase I (R&D Systems Inc.) was used as standard.

Statistical analyses were performed using commercially available software: SAS 9.2 (SAS Institute Inc., Cary, NC) for Receiver Operating Characteristic (ROC)/area under curve (AUC) calculations and logistic regression analyses, and SPSS 18 (SPSS Inc., Chicago, IL) for all other analyses.

Comparisons between CDR 0 and CDR 1 groups of the ‘discovery’ cohort (one sample was unavailable for re-evaluation, n = 47) were performed using unpaired t-test. For the ‘validation’ cohort (n = 292), correlations with age and gender were evaluated using the Spearman rho correlation coefficient (α = 0.05). Chi-square analyses were performed to evaluate need for adjustment for observed correlations. Comparisons between the three CDR groups were performed using one-way analysis of variance (ANOVA), with Bonferroni and LSD post-hoc tests for pair-wise group comparisons, with the following exceptions: one-way ANOVA with Welch's correction was applied for markers (transthyretin) demonstrating unequal variances (Levene <.05); markers correlating with age (tau, p-tau181, Aβ42, YKL-40) were evaluated by analysis of covariance (ANCOVA) adjusting for age, followed by Bonferroni and LSD post-hoc tests. Multiple post-hoc tests were applied in recognition of their different levels of stringency (Bonferroni > LSD), and their non-uniform popularity among statisticians. For CDR 0 vs >0 comparisons and CDR 1 vs <1 comparisons, unpaired t-test was used; Welch's correction for unequal variances was applied for YKL-40, p-tau181, tau, and Aβ42. For each biomarker measured in the larger ‘validation’ cohort, the ROC curve and the AUC were calculated for predicting CDR 0 versus CDR>0. A stepwise logistic regression analysis was used to identify an optimal combination of these biomarkers for this data set. These analyses were repeated for CDR 1 vs CDR<1.

## Results

### Sample Processing and 2D-DIGE Analysis

To identify new candidate biomarkers for AD, we utilized an unbiased proteomics approach, 2D-DIGE LC-MS/MS [32], [33], to compare the relative concentrations of CSF proteins in individuals with mild “probable AD” (CDR 1, n = 24) to those in individuals with normal cognition (CDR 0, n = 24). The two clinical groups were selected on the basis of relative biomarker values for CSF Aβ42 and tau (see Methods), and differed somewhat with respect to age at LP and gender (CDR 0: 64.8±8.8 yrs, 38% female; CDR 1: 72.8 yrs ±7.9 yrs, 54% female). Five samples showed evidence of blood contamination by 2D-DIGE; the five gels containing these samples were excluded from subsequent image analyses. The remaining individual sample images (n = 38, from 19 gels) were aligned using the BVA module (described under Methods).

**Figure 1:**
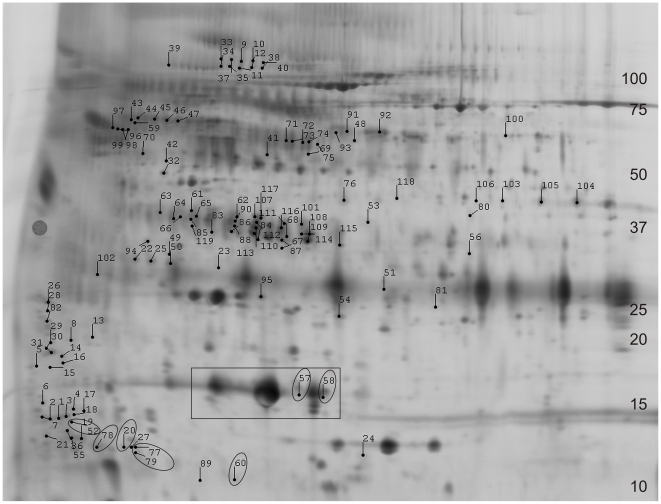
Two-dimensional difference in gel electrophoresis (2D-DIGE) of cerebrospinal fluid immunodepleted of six high abundance proteins. Representative 2D-DIGE (grayscale) image with labeled locations of 119 gel features that differed in intensity between CDR 0 and CDR 1 groups. Gel features are numbered 1 through 119, and relevant information about each is listed in Table 2 and in Table S1. Approximate molecular weight (in kilodaltons [kDa]) is indicated along the right border; isoelectric point ranges from 3 (left) to 11(right) and is non-linear (not shown). The large, intense, protein spots commonly attributed to transthyretin are boxed; a subset of the differentially abundant gel features in which transthyretin was identified by mass spectrometry is circled.

Among the 764 gel features that were present in >50% of the gels, 119 were found to have significant intensity differences between CDR 0 and CDR 1 groups (Student's t-test [α = 0.05]) (Figure 1). The image intensity data for these 119 gel features were subjected to unsupervised hierarchical clustering (EDA module, DeCyder software) and the gel features themselves were analyzed for protein composition.

**Table 2:** Proteins identified by 2D-DIGE LC-MS/MS with differential abundance in CDR 1 vs. CDR 0 CSF.

Spot	BVA	GI number(s)	Protein	Change	p value	Protein
**1**	4709	31543193	hypothetical protein LOC146556	−1.36	7.02E-04	1
**2**	5659	4502807	chromogranin B	−1.31	1.18E-03	2
**3**	4683	4502101	annexin I	−1.31	9.54E-04	3
**4**	4608	62089004	chromogranin B	−1.24	6.49E-03	
		181387	cystatin C			4
		134464	secretogranin-2			5
**5**	4297	4502807	chromogranin B	−1.26	0.0157	
**6**	4545			−1.34	3.86E-03	
**7**	4695	4502807	chromogranin B	−1.27	0.0115	
**8**	4044	4502807	chromogranin B	−1.32	2.15E-03	
**9**	1314	1621283	neuronal cell adhesion molecule (NrCAM)	−1.22	0.0119	6
**10**	1320	1621283	neuronal cell adhesion molecule (NrCAM)	−1.33	6.31E-04	
**11**	1382	6651381	neuronal cell adhesion molecule (NrCAM)	−1.28	9.53E-04	
**12**	1383	6651381	neuronal cell adhesion molecule (NrCAM)	−1.25	6.64E-03	
**13**	4033	4502807	chromogranin B	−1.21	0.0419	
**14**	4191	4502807	chromogranin B	−1.23	0.0107	
**15**	4293	4502807	chromogranin B	−1.33	4.64E-03	
		825635	calmodulin			7
**16**	4266	62089004	chromogranin B	−1.22	0.0315	
**17**	4615			−1.22	0.0188	
**18**	4677			−1.3	9.63E-03	
**19**	4906	5454032	S100 calcium binding protein A1	−1.3	1.36E-04	8
		62898141	prosaposin			9
		627391	brain-associated small cell lung cancer antigen/NCAM-140/CD56			10
		17136078	VGF nerve growth factor inducible precursor			11
**20**	5014	443295	transthyretin	−1.3	2.10E-03	12
**21**	4884	224917	apolipoprotein CIII	−1.34	9.78E-04	13
		337760	prosaposin/cerebroside sulfate activator protein			
**22**	3423	39654998	chain A, Hr1b Domain From Prk1	−1.27	0.0133	14
		32171249	prostaglandin H2 D-isomerase/beta trace			15
**23**	3470	17402888	neuronal pentraxin receptor	−1.25	7.23E-03	16
		114593356	extracellular superoxide dismutase (SOD3)			17
**24**	4954	34616	beta-2 microglobulin	−1.3	4.15E-03	18
**25**	3436	32171249	prostaglandin H2 D-isomerase	−1.22	0.0266	
		178775	proapolipoprotein			19
		39654998	chain A, Hr1b Domain From Prk1			
**26**	3714			−1.27	0.03	
**27**	4922	39654998	chain A, Hr1b Domain From Prk1	−1.27	0.0194	
**28**	3786	2072129	chromogranin A	−1.38	8.96E-03	20
**29**	4076	7341255	brain acetylcholinesterase putative membrane anchor	−1.25	0.0375	21
**30**	4111	62089004	chromogranin B	−1.28	0.0206	
**31**	4167	4502807	chromogranin B	−1.29	0.0207	
**32**	2652	28373309	gelsolin	−1.23	0.0346	22
**33**	1313	6651381	neuronal cell adhesion molecule (NrCAM)	−1.19	8.08E-03	
**34**	1372	1620909	ceruloplasmin	−1.19	9.00E-03	23
		1483187	inter-alpha-trypsin inhibitor family heavy chain-related protein			24
		31874098	hypothetical protein (NrCAM)			
		6651381	neuronal cell adhesion molecule (NrCAM)			
**35**	1387	68534652	neuronal cell adhesion molecule (NrCAM)	−1.29	8.16E-05	
		1620909	ceruloplasmin			
**36**	4808	337760	prosaposin/cerebroside sulfate activator protein	−1.22	0.0114	
**37**	1319	68534652	neuronal cell adhesion molecule (NrCAM)	−1.19	0.0198	
		1942284	ceruloplasmin			
**38**	1386	6651381	neuronal cell adhesion molecule (NrCAM)	−1.29	1.24E-03	
**39**	1353	21706696	calsyntenin 1	−1.22	0.0417	25
**40**	1329	1621283	neuronal cell adhesion molecule (NrCAM)	−1.22	4.61E-03	
**41**	2456	5802984	UDP-GlcNAc:betaGal beta-1,3-N-acetylglucosaminyltransferase 1	−1.13	0.0449	26
**42**	2550	20178323	pigment epithelium-derived factor precursor (PEDF)/Serpin-F1/EPC-1	−1.15	0.022	27
**43**	2125	21071039	carnosinase 1	−1.21	0.0245	28
**44**	2131	21071039	carnosinase 1	−1.19	0.049	
**45**	2152	21071039	carnosinase 1	−1.15	0.0366	
**46**	5614	21071039	carnosinase 1	−1.18	0.0109	
**47**	2166	21071039	carnosinase 1	−1.21	0.0122	
**48**	2328	416180	man9-mannosidase/α1,2-mannosidase IA	−1.16	0.0464	29
**49**	3360			−1.15	0.045	
**50**	3447	32171249	prostaglandin H2 D-isomerase/beta trace	−1.19	0.0334	
**51**	3546	1621283	neuronal cell adhesion molecule (NrCAM)	−1.17	0.0368	
		32171249	prostaglandin H2 D-isomerase/beta trace			
**52**	4745	443295	transthyretin	−1.26	0.0181	
**53**	3032	11056046	nectin-like molecule-1/SynCAM3/TSLL1	−1.13	0.0472	30
**54**	3718	39654998	chain A, Hr1b Domain From Prk1	−1.14	0.0455	
		32171249	prostaglandin H2 D-isomerase/beta trace			
**55**	4902	14277770	apolipoprotein C-Ii	−1.19	0.0495	31
		337760	prosaposin/cerebroside sulfate activator protein			
		2072129	chromogranin A			
**56**	3290	409725	carbonic anhydrase IV	−1.14	0.0141	32
**57**	4379	17942890	transthyretin	−1.15	0.0219	
		39654998	chain A, Hr1b Domain From Prk1			
		34999	cadherin 2 precursor			33
**58**	4388	32171249	prostaglandin H2 D-isomerase/beta trace	−1.14	0.0218	
		39654998	chain A, Hr1b Domain From Prk1			
		443295	transthyretin			
**59**	2192	21071039	carnosinase 1	−1.34	6.56E-03	
		532198	angiotensinogen			34
		5531817	secretogranin III			35
		9665262	EGF-containing fibulin-like extracellular matrix protein 1/Fibulin-3			36
		177933	alpha-1-antichymotrypsin			37
		4504893	kininogen 1			38
		36573	vitronectin			39
**60**	5336	443295	transthyretin	−1.17	0.0301	
**61**	3009	178855	apolipoprotein J/clusterin	−1.26	0.0288	40
		4557325	apolipoprotein E			41
**62**	3042	4557325/178853	apolipoprotein E	−1.21	0.047	
		338305	apolipoprotein J/clusterin			
**63**	3016	338305	apolipoprotein J/clusterin	−1.32	6.69E-05	
**64**	3050	4557325/178853	apolipoprotein E	−1.24	5.19E-04	
		178855	apolipoprotein J/clusterin			
**65**	3075	4557325/178853	apolipoprotein E	−1.42	5.59E-06	
		178855	apolipoprotein J/clusterin			
**66**	3038	4557325/178853	apolipoprotein E	−1.41	2.84E-05	
		178855	apolipoprotein J/clusterin			
**67**	3301	178849	apolipoprotein E	−1.4	1.29E-05	
**68**	3182	4557325/178853	apolipoprotein E	−1.41	3.43E-04	
		178855	apolipoprotein J/clusterin			
**69**	2443	532198	angiotensinogen	−1.2	6.85E-03	
**70**	2493	4503009	carboxypeptidase E precursor	−1.23	6.09E-03	42
**71**	5621	532198	angiotensinogen	−1.17	0.0434	
**72**	5624	532198	angiotensinogen	−1.22	0.0147	
**73**	5622	553181	angiotensinogen	−1.17	0.04	
**74**	5625	532198	angiotensinogen	−1.16	0.0423	
**75**	5627			−1.22	0.0113	
**76**	2849	4557325	apolipoprotein E	−1.28	6.26E-03	
**77**	5009	443295	transthyretin	−1.24	0.0268	
**78**	5033	443295	transthyretin	−1.27	4.59E-03	
**79**	5078	443295	transthyretin	−1.2	0.0144	
**80**	2958	4504067	aspartate aminotransferase 1	−1.22	8.60E-03	43
**81**	3657	32171249	prostaglandin H2 D-isomerase/beta trace	−1.22	3.07E-03	
**82**	3867			−1.28	0.0437	
**83**	3176	4557325	apolipoprotein E	−1.63	3.03E-04	
**84**	3228	4557325	apolipoprotein E	−1.4	1.39E-03	
		443295	transthyretin			
**85**	3074	4557325/178853	apolipoprotein E	−2.36	4.41E-09	
**86**	5647	4557325	apolipoprotein E	−2.35	2.92E-07	
**87**	3224	4557325/178853	apolipoprotein E	−2.13	6.36E-07	
		443295	transthyretin			
**88**	3126	4557325/178853	apolipoprotein E	−1.93	7.55E-06	
**89**	5297			−1.44	0.0473	
**90**	3083	4557325	apolipoprotein E	−1.7	2.82E-05	
**91**	2218	112911	alpha-2-macroglobulin	1.22	0.0282	44
**92**	2226	6573461	apolipoprotein H	1.27	0.0305	45
**93**	2252	112911	alpha-2-macroglobulin	1.26	0.0267	
		4557327	apolipoprotein H			
**94**	3255			1.24	0.0315	
**95**	3630	178775	proapolipoprotein	1.24	0.0287	
		32171249	prostaglandin H2 D-isomerase/beta trace			
		39654998	chain A, Hr1b Domain From Prk1			
**96**	2229	177933	alpha-1-antichymotrypsin	1.42	3.09E-03	
**97**	2235	177933	alpha-1-antichymotrypsin	1.35	0.0388	
**98**	2261	177933	alpha-1-antichymotrypsin	1.3	6.04E-03	
**99**	2262	177933	alpha-1-antichymotrypsin	1.25	0.0294	
**100**	2220			1.29	0.0158	
**101**	3084			1.16	0.0211	
**102**	3508	32171249	prostaglandin H2 D-isomerase/beta trace	1.22	9.21E-03	
**103**	2825	23512215	chitinase 3-like 1/YKL-40/HC-gp39	1.41	0.0167	46
**104**	2863	4557018	chitinase 3-like 1/YKL-40/HC-gp39	1.5	0.0144	
**105**	2846	29726259	chitinase 3-like 1/YKL-40/HC-gp39	1.46	7.88E-03	
**106**	2843	23512215	chitinase 3-like 1/YKL-40/HC-gp39	1.32	0.0241	
**107**	3030	4557325	apolipoprotein E	2.46	3.70E-05	
**108**	3152	4557325/178853	apolipoprotein E	2.39	8.73E-07	
**109**	3203	178853	apolipoprotein E	3.23	3.13E-07	
**110**	3185	4557325/178853	apolipoprotein E	1.9	9.72E-04	
		443295	transthyretin			
**111**	3069	338305	apolipoprotein J/clusterin	1.5	6.40E-04	
**112**	3079			1.64	4.47E-04	
**113**	3133	178853	apolipoprotein E	1.49	8.66E-04	
		338057	apolipoprotein J/clusterin			
**114**	3151	178853	apolipoprotein E	1.28	9.25E-03	
		338057	apolipoprotein J/clusterin			
**115**	3249	4557325	apolipoprotein E	1.37	2.46E-03	
		178855	apolipoprotein J/clusterin			
		443295	transthyretin			
**116**	3118	4557325/178853	apolipoprotein E	1.64	9.96E-04	
**117**	5698	178855	apolipoprotein J/clusterin	1.73	5.82E-04	
**118**	2819	40737343	C4B3	2	0.038	47
**119**	3137	4557325	apolipoprotein E	−2.5	8.52E-07	

Column 1, coded protein spot ID (as in Figure 1).

Column 2, biological variation analysis (BVA) number for spot generated by Decyder software.

Column 3, GI accession number(s) assigned to proteins identified by MASCOT.

Column 4, name of protein identified by MASCOT.

Column 5, fold-change in protein abundance; negative values indicate decreases in CDR 1 vs. CDR 0.

Column 6, p value of the CDR 1 versus CDR 0 comparison (Student's t test).

Column 7, consecutive numbering identifying proteins as unique.

### Protein Identification by LC-MS/MS

LC-MS/MS identified single dominant proteins in 78 of the 119 gel features (Table 2). In 29 gel features, our analyses identified two or more co-dominant proteins. The 12 remaining gel features were not annotated from the nano-LC-MS/MS data. Among the characterized gel features, there was considerable redundancy in protein identifications, with some proteins appearing in multiple gel features. Such ‘redundant’ gel features, likely representing a modified form or variant of the same ‘parent’ protein, generally migrated with some proximity on 2D-gel electrophoresis (Figure 1). Forty-seven unique proteins were identified (Table 2). Thirteen of these unique proteins had been identified in our previous studies [32], [33] (including chromogranin B, cystatin C, prostaglandin H2 D-isomerase/beta trace, neuronal pentraxin receptor, gelsolin, beta-2 microglobulin, carnosinase I, angiotensinogen, apolipoprotein H, secretogranin III, alpha-1-antichymotrypsin, chitinase 3-like 1/YKL-40, and kininogen I) and others have been reported by other groups [17], [19], [20], [23], [25], [27]. These previous reports provide supporting evidence that this list of proteins may contain viable candidate biomarkers for AD that are worthy of pursuit in validation experiments.

### Unsupervised Clustering Analysis

**Figure 2:**
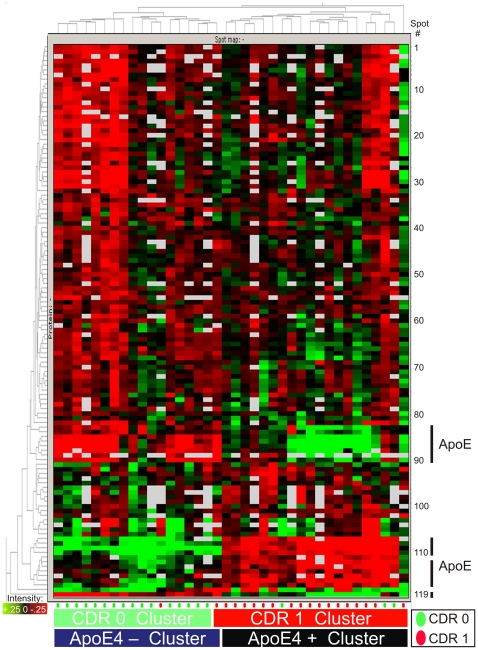
Unsupervised clustering of CSF samples by 2D-DIGE data from the 119 statistically significant gel features. (Student's t-test, α = 0.05, present in >50% of images). Five gels containing hemoglobin (n = 10 samples) were excluded. Columns represent samples; rows, numbered 1 through 119 from top to bottom, represent gel features depicted in Figure 1. Gel feature intensity is encoded colorimetrically from red (low intensity) to green (high intensity); white indicates absent data. CDR status of individuals at time of CSF collection is encoded below by small green (CDR 0) and red (CDR 1) ovals; CDR 0 and CDR 1 clusters are indicated below by green and red bars, respectively. *APOE-ε4* allele status of individuals and groups, alike, is indicated by black (possessing ApoE4 protein, or one or two *APOE-ε4* alleles) or blue (possessing no ApoE4 protein, or no *APOE-ε4* alleles) bars. Rows representing gel features containing ApoE protein are indicated along the lower right border.

**Figure 3:**
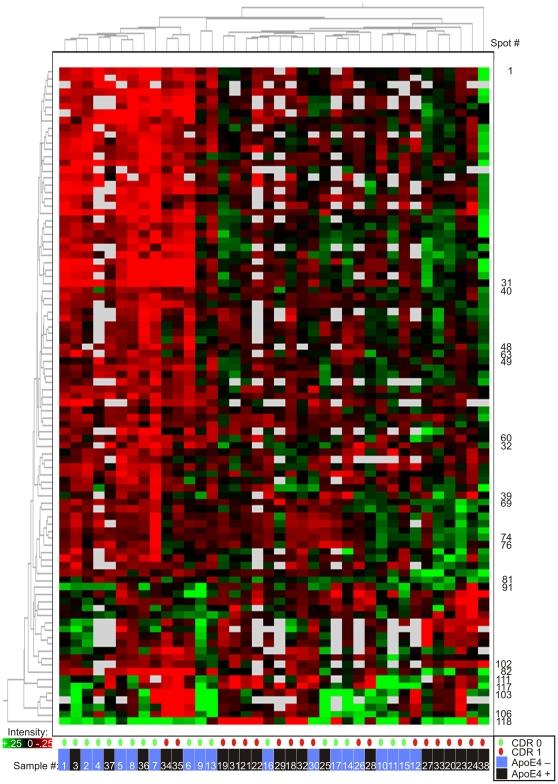
Unsupervised clustering of CSF samples by 2D-DIGE data, excluding gel features containing apoE protein. All other statistically significant gel features (Student's t-test α = 0.05, present in >50% of images) are retained. As in Figure 2, five gels containing hemoglobin (n = 10 samples) were excluded. Columns represent samples, numbered according to their original positions in Figure 2. Rows represent gel features, numbered as in Figure 2; unlabeled rows are in consecutive order from upper number to lower number, with interruptions in sequence indicated by labels. ApoE-containing features are removed. Gel feature intensity is encoded colorimetrically from red (low intensity) to green (high intensity); white indicates absent data. CDR status of participants at time of CSF collection is encoded below, by small green (CDR 0) and red (CDR 1) ovals. *APOE-ε4* status (as described for Figure 2) is indicated by blue (ApoE4 negative) or black (ApoE4 positive) bars, below. Clustering pattern of samples (numbered consecutively in order of appearance in Figure 2, from left to right) relative to Figure 2 is indicated by white numerals, below.

The intensity data from the 119 gel features of interest were subjected to an unsupervised clustering analysis to evaluate their ability to segregate the CDR 0 and CDR 1 samples, and to assess their collective potential as a diagnostic biomarker panel (Figure 2). The ‘heatmap’ generated from this analysis appeared to segregate CDR 0 and CDR 1 individuals (indicated by green and red ovals, respectively) almost completely, with only four participants ‘misclassified.’ However, closer examination revealed an additional layer of segregation on the basis of *APOE* genotype (indicated by ‘ApoE 4+ Cluster’ and ‘ApoE 4 – Cluster’) which showed perfect sample segregation. Given that the *APOE-ε4* allele is a dominant genetic risk factor for AD, some clustering of individuals by *APOE* genotype might be expected simply from successful segregation of CDR 0 and CDR 1 individuals. However, we hypothesize that the apoE protein exerts a dominant clustering influence through the markedly different electrophoretic profiles of its different isoforms derived from *APOE-ε2*, *APOE-ε3* and *APOE- ε4* alleles (illustrated in Figure S1). ApoE was present in 24 of the 119 gel features found to differ in intensity between the CDR groups, and was found to be the primary protein in 12 of these gel features. This heterogeneous electrophoretic mobility of apoE results from the inherent charge differences of the three major apoE isoforms (-E2, -E3, -E4) and the appearance of each isoform as an array of multiple distinct gel features caused by post-translational modifications. These isoform-specific differences are reflected in the prominent red and green clusters, located within the lower third of Figure 2 (corresponding to gel features 83–90, 107–117, and 119), that correlate very closely with participant *APOE* genotypes. Recognizing this correlation, we hypothesized that *APOE* genotypes were in large part driving the clustering of participant samples in Figure 2. To test this hypothesis, we performed a second unsupervised clustering analysis, including only those gel features from the initial analysis that did not contain apoE protein (Figure 3). Although this ‘apoE-free’ analysis segregated CDR 1 and CDR 0 groups less completely, it appropriately re-clustered (by CDR status) several samples (#12, 36, 37) that were aberrantly segregated in Figure 2, potentially due to their *APOE* genotypes. Moreover, clustering of participant samples into *APOE* genotype subgroups in Figure 3 appears negligible. The underlying benefit of this ‘apoE-free’ analysis is that it reveals the sample-clustering potential of other gel features, which was previously obscured by the inclusion of apoE-containing gel features. As can now be better visualized in Figure 3, gel features appearing within the upper three-fourths of the heatmap appear to show greater intensity in CDR 1 samples; the converse is true of gel features within the lower fourth. It is important to note that measurements of Aβ42 and tau (two proteins measured by ELISA and not detected by 2D-DIGE) were not included in these clustering analyses; because these ‘discovery’ samples were selected for this study on the basis of CSF Aβ42 and tau levels, such inclusion would presumably yield perfect or near-perfect segregation by CDR status in this ‘discovery’ cohort. Therefore, this analysis reflects the potential of these candidate biomarkers to segregate CDR 0 and CDR 1 individuals *independent* of any contribution from current leading CSF biomarkers Aβ42 and tau. It does not address whether these biomarker candidates might improve upon the utility of Aβ42 and tau, however.

### Validation of Candidate Biomarkers by ELISA

Before evaluating a subset of these candidate biomarkers in a larger independent sample set, we first assessed the capacity of protein-specific quantitative ELISAs to detect significant differences between the CDR 0 and CDR 1 groups of the original ‘discovery’ cohort. When possible, to facilitate future reproduction of our findings by other groups and potential translation to clinical use, we applied commercially available ELISA kits.

**Figure 4:**
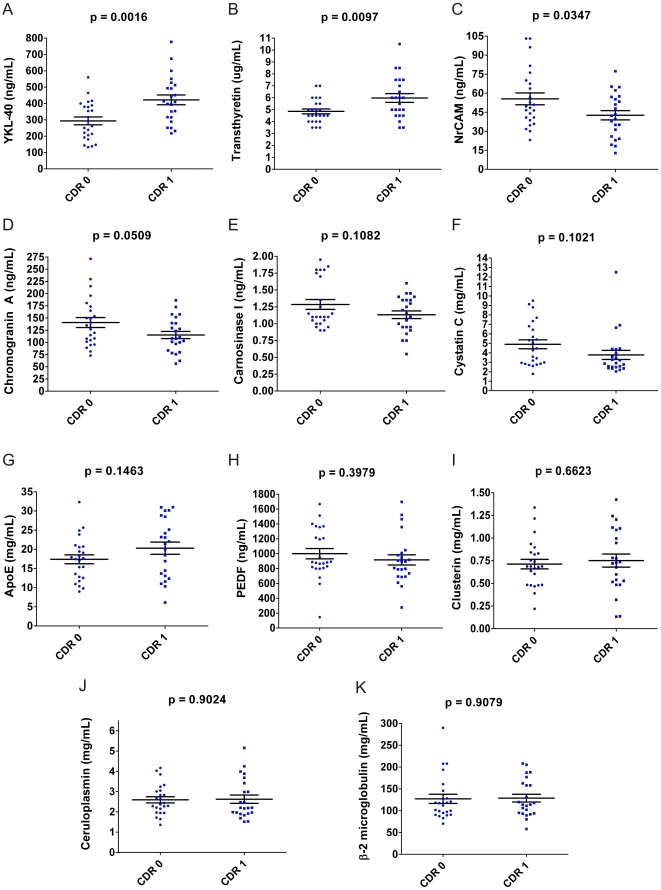
Quantitative ELISAs for 11 biomarker candidates applied to ‘discovery’ cohort CSF samples (n = 47). Each assay performed in triplicate; mean value reported for each sample. The six assays represented in the upper two rows (**A**. YKL-40, **B**. Transthyretin, **C**. NrCAM, **D**. Chromogranin A, **E**. Carnosinase I, and **F**. Cystatin C) measured differences between CDR 0 and CDR 1 groups (unpaired t-test); the five assays represented in the lower two rows (**G**. ApoE, **H**. PEDF, **I**. Clusterin, **J**. Ceruloplasmin, **K**. β-2 microglobulin) did not.

Of the eleven ELISAs applied to the ‘discovery’ cohort (n = 47, one sample was unavailable for validation), six (NrCAM, YKL-40, chromogranin A, carnosinase I, transthyretin, cystatin C) showed statistically significant or near-significant differences between CDR 0 and CDR 1 groups (Figure 4); five others (PEDF, beta-2 microglobulin, clusterin/apoJ, ceruloplasmin, apoE) did not.

The six ELISAs that measured differences between the CDR 0 and CDR 1 CSF samples of the ‘discovery’ cohort were subsequently applied to a larger, independent set of CSF samples (n = 292) collected from volunteer participants studied by the WU-ADRC. This ‘validation’ cohort included a CDR 0.5 group in addition to CDR 0 and CDR 1 groups, allowing for biomarker assessment in the very early clinical stage of AD. Demographic, clinical, and genetic characteristics of these individuals at time of sample collection are presented in Table 1. Unlike the ‘discovery’ cohort, this ‘validation’ cohort was not preselected on the basis of prior biomarker values (CSF Aβ42 and tau), although assays for CSF Aβ42, tau and p-tau181 were performed.

Because the age and gender compositions differed among the clinical groups of the ‘validation cohort,’ we evaluated each of these 9 biomarkers (six novel candidates, Aβ42, tau, and p-tau181) for age and gender correlations in order to apply covariate analyses appropriately. Correlating with age were tau (r = 0.318, p<0.0001), p-tau181 (r = 0.2216, p<0.001), Aβ42 (r = −0.2334, p<0.0001) and YKL-40 (r = 0.4001, p<0.001); no biomarkers correlated with gender (p>0.05).

**Figure 5:**
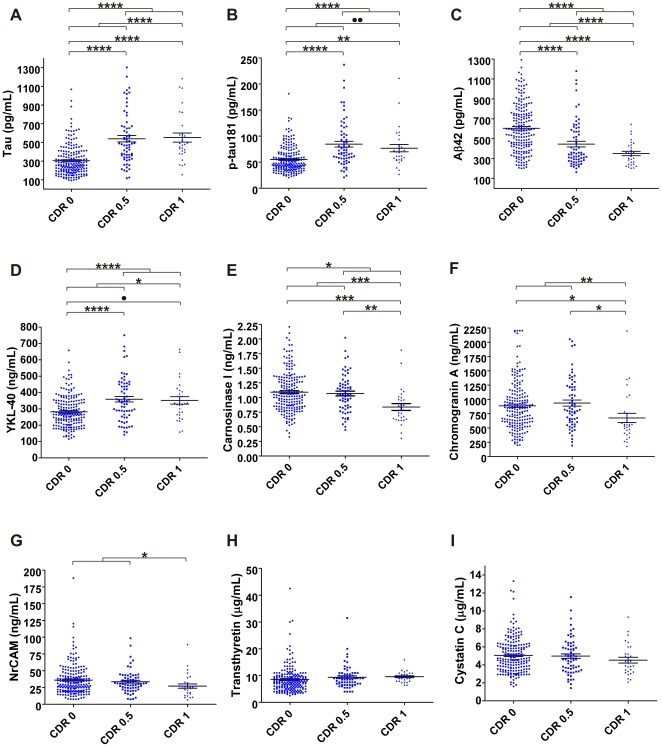
Six biomarker candidates and established biomarkers tau, p-tau181 and Aβ42 in ‘validation’ cohort CSF (n = 292). Each candidate biomarker assay was performed in triplicate, with one mean value reported for each sample; assays for tau, p-tau181 and Aβ42 were performed in duplicate. In addition to **A**. tau, **B**. p-tau181 and **C**. Aβ42 (top row), four assays (**D**. YKL-40, **E**. carnosinase I, **F**. chromogranin A, **G**. NrCAM) measured statistical differences between clinically defined groups, as indicated; **H**. transthyretin and **I**. cystatin C did not reach criterion (α = 0.05) for any comparisons. * p<0.05; * * p<0.01; * * * p< 0.001; * * * * p<0.0001; solid circle p<0.05 by LSD only; **double solid circle p<0.05** by unpaired t-test and Mann-Whitney, not by unpaired t-test with Welch's correction.

As shown in Figure 5, statistically significant differences between clinically defined groups were measured for Aβ42, tau, p-tau181, NrCAM, YKL-40, chromogranin A, and carnosinase I; for transthyretin and cystatin C, non-significant trends were measured. These differences appeared in three patterns: Aβ42 showed a pronounced decrease from CDR 0 to CDR 0.5 and a lesser reduction from CDR 0.5 to CDR 1; tau, p-tau181, and YKL-40 showed increases that were equivalent in CDR 0.5 and CDR 1 relative to CDR 0; NrCAM, chromogranin A, and carnosinase I showed decreases relative to CDR 0 only in CDR 1, and not in CDR 0.5.

### Diagnostic Utility of Validated Candidate Biomarkers

**Figure 6:**
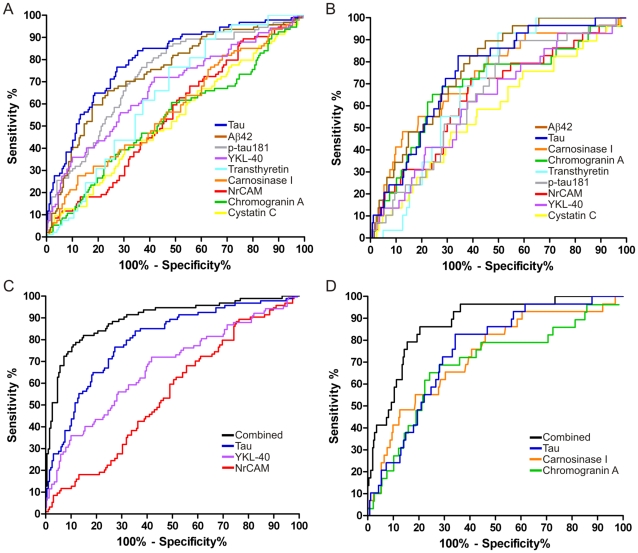
Receiver Operating Characteristic (ROC) curves of ELISA data from ‘validation’ cohort. Simple ROC analyses were performed for each biomarker to distinguish **A**. CDR>0 from CDR 0 (“earlier diagnosis”) and **B**. CDR 1 from CDR<1 (“early diagnosis”). Stepwise logistic regression models were used to identify combinations of these biomarkers that would distinguish **C**. CDR>0 from CDR 0 (“earlier diagnosis”), AUC = 0.90 and **D**. CDR 1 from CDR<1 (“early diagnosis”), AUC = 0.88.

To evaluate and compare the potential of the validated candidate biomarkers and Aβ42, tau, and p-tau181 for identifying either very mild to mild dementia (combined CDR 0.5 and CDR 1) or mild dementia (CDR 1), ROC curves and AUCs were calculated for each biomarker using data from the ‘validation’ cohort (Figure 6A, B, Tables 3, 4). Stepwise logistic regression analyses indicated that, among the nine biomarkers under consideration, YKL-40, NrCAM and tau yielded the highest AUC (0.896) in discriminating cognitive normalcy (CDR 0) from very mild to mild dementia (CDR>0) (Figure 6C, Table 3); for discriminating mild dementia (CDR 1) from CDR<1, carnosinase I, chromogranin A and tau yielded the highest AUC (0.876) (Figure 6D, Table 4).

**Table 3:** Receiver Operating Characteristic Curve Areas for CDR 0 vs <0 Comparison.

Biomarker	Area Under Curve	Standard Error	95% Confidence Interval
Tau	0.8004	0.0279	0.7457–0.8551
Aβ42	0.7429	0.0315	0.6812–0.8046
p-tau181	0.7339	0.0315	0.6721–0.7956
YKL-40	0.6717	0.0349	0.6033–0.7401
Transthyretin	0.6190	0.0331	0.5541–0.6838
Carnosinase I	0.5735	0.0365	0.5020–0.6450
NrCAM	0.5422	0.0355	0.4726–0.6118
Chromogranin A	0.5303	0.0373	0.4572–0.6034
Cystatin C	0.5297	0.0366	0.4579–0.6014
Logistic Regression	0.8955	0.0212	0.8539–0.9372

ROC analyses of ‘validation’ cohort ELISA data were performed for each biomarker to distinguish CDR>0 from CDR 0 (“earlier diagnosis”). A stepwise logistic regression model, applied to identify a complementary combination of these biomarkers that would optimize accuracy (maximize area under the curve [AUC]) without including additional non-contributory biomarkers, accepted tau, YKL-40 and NrCAM and yielded an AUC of 0.8955 (“Logistic Regression,” lowest row).

**Table 4:** Receiver Operating Characteristic Curve Areas for CDR 1 vs <1 Comparison.

Biomarker	Area Under Curve	Standard Error	95% Confidence Interval
Aβ42	0.7690	0.0376	0.6953–0.8427
Tau	0.7502	0.0420	0.6679–0.8325
Carnosinase I	0.7277	0.0512	0.6273–0.8281
Chromogranin A	0.6879	0.0566	0.5771–0.7988
Transthyretin	0.6605	0.0380	0.5860–0.7350
p-tau181	0.6512	0.0483	0.5566–0.7458
NrCAM	0.6411	0.0553	0.5326–0.7495
YKL-40	0.6271	0.0532	0.5228–0.7313
Cystatin C	0.5752	0.0565	0.4645–0.6858
Logistic Regression	0.8762	0.0314	0.8147–0.9377

ROC analyses of ‘validation’ cohort ELISA data were performed for each biomarker to distinguish CDR 1 from CDR<1 (“early diagnosis”). A stepwise logistic regression model, applied to identify a complementary combination of these biomarkers that would optimize accuracy (maximize area under the curve [AUC]) without including additional non-contributory biomarkers, accepted tau, carnosinase I and chromogranin A, yielding an AUC of 0.8762 (“Logistic Regression,” lowest row).

## Discussion

Using an unbiased proteomics approach (2D-DIGE LC-MS/MS), this study identified 47 novel candidate CSF protein biomarkers for early AD. Subsequently, by evaluating a subset of these candidate biomarkers by ELISA, this study validated the utility of four candidate biomarkers for distinguishing groups with mild, very mild, or no dementia (CDR 1, 0.5, 0, respectively). Further statistical analyses demonstrated that these biomarkers could improve the accuracy of ‘established’ biomarkers Aβ42 and tau for the diagnosis of early AD.

The results from the 2D-DIGE LC-MS/MS portion of this study suggest that many of the recognized neuropathological changes of AD are represented by changes in the CSF proteome. Most of the 47 candidate biomarker proteins identified in this study can be placed into structural and/or functional categories (e.g. synaptic adhesion, synaptic function, dense core synaptic vesicle proteins, inflammation/complement, protease activity/inhibition, apolipoproteins, etc.) associated with accepted neuropathophysiological changes in AD (Table 5). Unsupervised clustering analyses of these 2D-DIGE data, performed without the influence of CSF Aβ42, tau, p-tau181 and *APOE* genotype, additionally suggest that these biomarker candidates collectively show utility for discriminating groups with and without mild DAT (Figure 3).

**Table 5:** Candidate CSF biomarkers reflect AD-related pathophysiologic changes.

Functional/Structural Category	Protein	References
Adhesion molecules	N-Cadherin	[39]-[45]
	NrCAM	[19], [46]-[49]
	Calsyntenin	[47], [50]-[53]
	Neuronal Pentraxin Receptor	[47], [54]
	Brain Associated Small Cell Lung Cancer Antigen (NCAM-140/CD56)	[55]
	Nectin-like molecule-1/TSLL1/SynCam3	[56]-[58]
Dense core vesicles	Chromogranin A	[19], [20], [22], [59]-[62]
	Chromogranin B	[60], [62]
	Secretogranin II	[60]-[63]
	Secretogranin III	[59], [64], [65]
	VGF NGF Inducible precursor	[20], [22], [23], [66]-[69]
	Carboxypeptidase E	[70]-[75]
Synaptic/Neuronal metabolism	Aspartate aminotransferase I	[76]-[82]
Synaptic Function	S100A1	[83]
	Neuronal Pentraxin Receptor	[27], [47], [54]
	Brain Acetylcholinesterase Putative Membrane Anchor (CutA1)	[84], [85]
	Calsyntenin	[47], [50]-[53]
Neuroprotection	PEDF (Serpin-F1)	[86]-[96]
	Annexin I	[97]-[99]
	Prosaposin	[20], [100]-[103]
	Secretogranin II	[104]-[106]
	Carnosinase I	[33], [107]-[111]
	Extracellular superoxide dismutase (SOD3)	[112]-[114]
Apoptosis/Actin remodeling	Gelsolin	[115]-[121]
	Prk-1 (PKN)	[122]-[126]
Synaptic plasticity/Learning and memory	VGF NGF inducible precursor	[20], [22], [23], [66]-[69]
	NrCAM	[19], [46]-[49]
	β3GnT1	[49], [127], [128]
	Carnosinase I	[33], [107]-[111]
	Carbonic Anhydrase IV	[129]-[131]
	S100A1	[132]
	Carboxypeptidase E	[70]-[75]
	Calmodulin	[133]-[136]
	Extracellular superoxide dismutase (SOD3)	[114]
Inflammation/Complement	*YKL-40/Chitinase 3-Like 1	[137]-[148]
	PEDF (Serpin-F1)	[86]-[96]
	Annexin I	[97]-[99]
	IHRP/ITIH4	[149], [150]
	Vitronectin	[151]-[155]
	*Complement C4B3	[156]-[161]
	Kininogen I	[162], [163]
	Chromogranin A	[19], [20], [22], [59]-[62]
	Secretogranin III	[59], [64], [65]
	Apolipoprotein J	[27], [152], [156], [157], [164]-[167]
	Beta 2-microglobulin	[168]-[171]
	Extracellular superoxide dismutase (SOD3)	[172]
Prostaglandin metabolism	*Prostaglandin H2 D Isomerase/Beta-trace	[162], [173]-[175]
Amyloid beta peptide binding/Amyloidogenesis	*Apolipoprotein A1 (proapolipoprotein)	[176], [177]
	Apolipoprotein E	[178]
	Apolipoprotein J	[27], [152], [156], [157], [164]-[167]
	Transthyretin	[87], [173], [175], [179]-[184]
	Gelsolin	[115]-[121]
	Vitronectin	[151]-[155]
	Cystatin C	[22], [173], [185]-[188]
	*Prostaglandin H2 D Isomerase/Beta-trace	[162], [173]-[175]
	*α-2-macroglobulin	[19], [189]-[194]
	*α-1-antichymotrypsin	[33], [195]-[199]
Protease activity	*α-1-antichymotrypsin	[33], [195]-[199]
	*α-2-macroglobulin	[19], [189]-[194]
	Cystatin C	[22], [173], [185]-[188]
	Carboxypeptidase E	[70]-[75]
Matrix proteins	Fibulin 3 (EFEMP1)	[200]-[202]
	Vitronectin	[151]-[155]
Phospholipase activity	Annexin I (Lipocortin)	[97]-[99]
	Prosaposin	[20], [100]-[103]
Apolipoproteins	*Apolipoprotein A1 (proapolipoprotein)	[24], [165], [166], [176], [177], [203], [204]
	Apolipoprotein CII	[25], [166], [205]
	Apolipoprotein CIII	[25], [206], [207]
	Apolipoprotein E	[24], [27], [165], [204]
	Apolipoprotein J	[27], [152], [156], [157], [164]-[167]
	*Apolipoprotein H	[19], [25], [165], [208], [209]
Calcium binding/homeostasis	Calmodulin	[134]-[136]
	S100A1	[83], [210]
	Annexin I (Lipocortin)	[97]-[99]
	Calsyntenin	[47], [50]-[53]
	Gelsolin	[115]-[121]
Metal (Copper and Iron) Binding	Carnosinase I	[33], [107]-[111]
	Ceruloplasmin	[211]-[217]
	Brain Acetylcholinesterase Putative Membrane Anchor (CutA1)	[84], [85]
Chaperone complex/activity	S100A1	[218]
	Transthyretin (prealbumin)	[24], [87], [173], [175], [179]-[184]
Endoplasmic Reticulum - Associated Degradation	Man9-mannosidase	[219]-[221]
Extracellular and Intraneuronal pH	Carbonic Anhydrase IV	[129]-[131]
	Carnosinase I	[33], [107]-[111]
Glycobiology (lactosamine synthesis)	β3GnT1	[49], [127], [128]
Hemodynamics	Angiotensinogen	[172], [222]
	Extracellular superoxide dismutase (SOD3)	[172]
Thyroid hormone transport	Transthyretin (prealbumin)	[24], [87], [173], [175], [179]-[184]
Unknown	Hypothetical protein	

CSF biomarkers are grouped according to reported function(s) and, when appropriate, cellular locations. Asterisks (*) indicate those biomarkers found to be increased in AD CSF; the vast majority were decreased.

In the second phase of this study, designed to measure a subset of candidate biomarker proteins in two independent sample sets by ELISA, four of the eleven candidate biomarkers that were tested showed capacity to distinguish clinical groups. However, seven candidate biomarkers did not show statistically significant differences between clinical groups in either the smaller ‘discovery’ cohort or the larger ‘validation’ cohort. Superficially, this ‘failure rate’ might cast doubt on the list of candidate biomarkers identified through 2D-DIGE. However, it is important to note that 2D-DIGE is sensitive to changes in concentrations of minor protein isoforms and post-translational modifications that may not significantly alter the global concentrations of a ‘parent’ protein, which would be measured by ELISA. Therefore, it is not surprising that some of the candidate biomarker ELISAs did not replicate the findings from 2D-DIGE. Transthyretin provides a prime example: all of the significant gel-features ascribed to transthyretin (gel features # 20, 52, 57, 58, 60, 77, 78, 79, 84, 87, 110, 115; Table 2) showed unusual electrophoretic patterns and were dwarfed by the canonical transthyretin gel features that did not individually show statistical differences (Figure 1). In fact, whereas most of the significant transthyretin 2D-DIGE gel features were decreased in AD, the global transthyretin levels measured by ELISA in the ‘discovery’ and ‘validation’ cohorts were actually mildly increased in groups with cognitive impairment (CDR>0) relative to those without (CDR 0) (Figures 4 and 5). To measure the sub-species of transthyretin that were identified by 2D-DIGE as decreasing in AD will require assays that specifically target relevant post-translational modifications and exclude other forms of transthyretin. Similarly, other 2D-DIGE biomarker candidates may also require specifically tailored assays for accurate, high-throughput measurement.

Nevertheless, four candidate biomarkers were successfully validated in both cohorts, and two others showed non-significant trends by ELISA in the larger ‘validation’ cohort (Figure 5). This larger cohort represented three different cognitive stages: normalcy, very mild dementia, and mild dementia (CDR 0, CDR 0.5, CDR 1, respectively), and revealed different patterns of CSF biomarker levels, *vis-a-vis* cognitive status. The CSF concentration of YKL-40, an astrocytic marker of plaque-associated neuroinflammation [137]–[148], is increased by the very earliest stage of clinical disease (CDR 0.5). Transthyretin [24], [87], [173], [175], [179]–[184] and cystatin C [22], [173], [185]–[188], two proteins with neuroprotective qualities that are implicated in preventing amyloidogenesis of Aβ peptide, show a similar pattern. In contrast, the concentrations of NrCAM, a synaptic adhesion molecule [19], [46]–[49], chromogranin A, a dense core synaptic vesicle protein [19], [20], [22], [59]–[62], and carnosinase I, a neuronal dipeptidase responsible for degradation of the anti-oxidant and metal-chelating dipeptide carnosine [33], [107]–[111] do not decline until mild dementia ensues (CDR 1).

Like the current leading CSF biomarkers for AD (Aβ42, tau and p-tau181), all of these biomarker candidates show ranges with substantial overlap between clinically defined groups. This issue of overlapping values, common among candidate AD CSF biomarkers reported to date, suggests that any one biomarker will be insufficient to accurately identify early AD, and that an ensemble of complementary biomarkers will be required to provide adequate sensitivity and specificity. Therefore, to identify an optimal combination of these biomarkers that can distinguish the early clinical stages of AD from cognitive normalcy, we applied stepwise logistic regression analyses to the ELISA data from our ‘validation’ cohort (Figure 6, Tables 3 and 4). These analyses suggest that four candidate AD biomarkers (YKL-40, NrCAM, chromogranin A, carnosinase I) can improve the ability of tau to classify individuals into CDR 0, CDR 0.5 and CDR 1 groups with appreciable accuracy.

It may appear counter-intuitive that Aβ42 and p-tau181, which individually discriminate very mild AD and mild AD from cognitively normal groups quite well, were not incorporated into either ‘optimal’ biomarker panel by the stepwise logistic regression analyses. Likewise, NrCAM was included in the optimal CDR 0 vs CDR>0 biomarker panel (AUC 0.896) even though its mean levels did not independently show a statistical difference between CDR 0 and CDR>0 groups. In considering this outcome, it may be worth noting that if NrCAM, transthyretin, chromogranin and cystatin C are removed from consideration, the stepwise logistic regression model for the CDR 0 vs CDR>0 comparison yields an ‘optimal’ biomarker panel that includes only tau, Aβ42 and carnosinase I, with an AUC of 0.849 (not shown). In this restricted analysis, the paired contribution of Aβ42 and carnosinase I to tau is apparently greater than that of YKL-40. These analyses illustrate how ‘unpredictable’ and context-dependent optimal biomarker combinations can be, and suggest that biomarker complementarity may be more important to consider than each biomarker's independent performance, when choosing a biomarker panel. Of course, it will be necessary to replicate these findings in additional independent cohorts. It will also be essential to evaluate a greater number of candidate biomarkers in similar fashion, in order to construct a biomarker panel with even greater accuracy.

Another worthwhile feature to consider when evaluating and selecting CSF biomarkers is relative concentration in the blood (plasma, serum), because biomarker measurements in CSF can be artifactually influenced by subtle blood contamination at the time of lumbar puncture; from this perspective, ideal CSF biomarkers show CSF concentrations that are equal to or greater than those in blood. An additional reason to assess plasma/serum concentrations of candidate CSF biomarkers is to determine if venipuncture, which is more easily performed than lumbar puncture, might yield equivalent information. Among the six CSF biomarkers identified by stepwise logistic regression analysis in the current study, Aβ42 and tau [8]–[11], YKL-40 [137], and chromogranin A [223] show higher levels in CSF than in plasma; carnosinase I levels appear similar in CSF and serum [110]; NrCAM levels appear higher in serum than in CSF, although the forms of NrCAM present in these fluids may differ [224]. Concerning independent utility as biomarkers for AD, only plasma YKL-40 and serum NrCAM have shown promise [137], [225], albeit inferior to that of CSF YKL-40 and NrCAM demonstrated here. Plasma tau concentrations in AD and controls are below the level of detection of the most commonly used tau assays, and plasma Aβ42 [8]–[11] and plasma chromogranin A (R.Perrin et al., unpublished data) concentrations show no significant differences among CDR groups. Serum carnosinase *activity* likewise has not shown significant differences between AD and controls in one small study [111], though a difference between AD and mixed dementia (including vascular dementia) has been reported [111]. To our knowledge, an evaluation of plasma or serum carnosinase I *concentrations* in the context of AD has not yet been performed or reported. Further assessment of the potential of these and other proteins as candidate AD biomarkers in plasma or serum, complete with evaluation of their performance as ensembles, remains an important task for future studies. Currently, however, this panel of six biomarkers appears likely to show much greater promise in its application to CSF.

**Figure 7:**
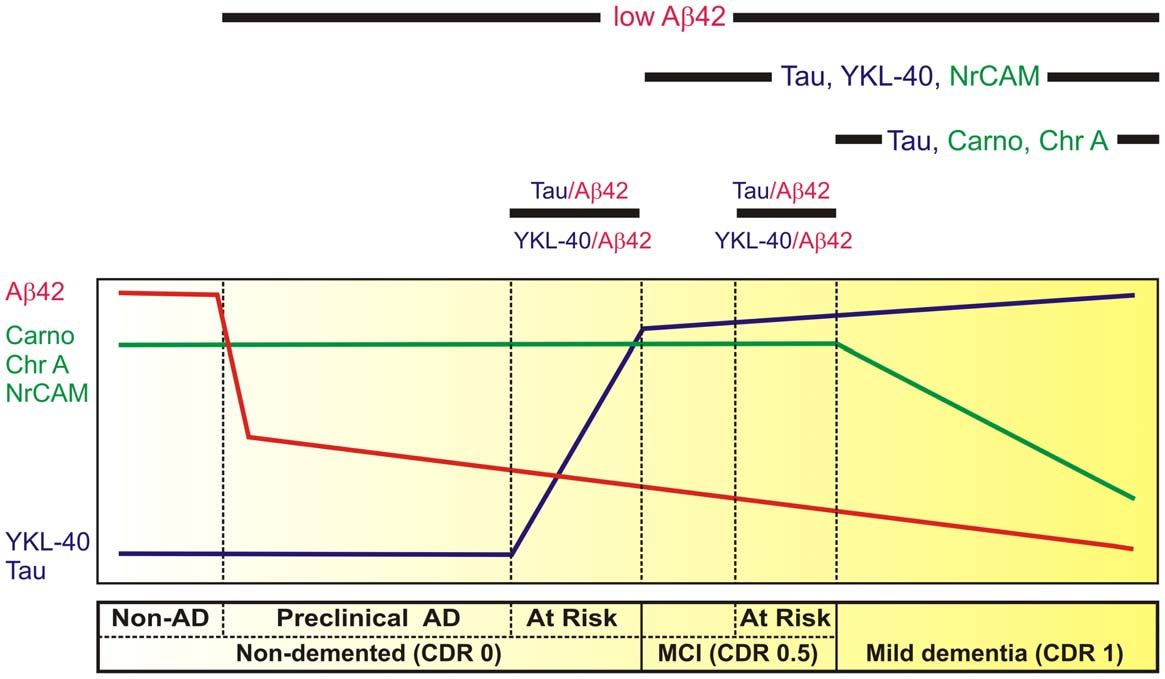
Hypothetical model defines early stages of AD by temporal pattern of CSF protein biomarker levels. The horizontal bar (below) describes the early clinicopathological progression from cognitive normalcy without AD pathology (‘**Non-AD**’) to mild dementia in six stages. As depicted by the curves above, **Non-AD** CSF has high Aβ42 (red line), high chromogranin A (Chr A), carnosinase I (Carno I) and NrCAM (green line), and low YKL-40 and tau (blue line). Reduced CSF Aβ42 correlates with amyloid plaque deposits, the first sign of neuropathologically identifiable AD (‘**preclinical AD**’) [8]. CSF Aβ42 appears to decrease further as cognition declines from normal (Clinical Dementia Rating [**CDR**] **0**) to very mild cognitive impairment (**MCI, CDR 0.5**) to mild dementia (**CDR 1**). When considered as ratios with Aβ42, CSF markers of neuroinflammation (e.g. YKL-40) and neurofibrillary tangle pathology (e.g. tau) appear to increase before and predict the onset of very mild cognitive impairment (**MCI, CDR 0.5**), defining a **CDR 0** group **‘At Risk’** for cognitive decline [9], [15], [137]; YKL-40 and tau also appear to be higher among those who progress rapidly from very mild to mild dementia, defining a **CDR 0.5** group **‘At Risk’** for impending cognitive decline [137], [230]. Reductions in synapse-associated (NrCAM, chromogranin A) and neuronal (carnosinase I) proteins, and increases in YKL-40 and tau mirror the progression and anatomical spread of synaptic and neuronal losses, gliosis and tau pathology associated with cognitive decline, and can be used to define **CDR 0.5** and **CDR 1**.

Indeed, by providing proof of concept, this study outlines a scheme to categorize the early stages of AD using CSF protein biomarkers that reflect established features of the pathophysiological evolution of the disease (Figure 7). Building upon previous findings that low CSF Aβ42 can identify cognitively normal individuals with plaques (preclinical AD) [8], [11], and that tau/Aβ42 and YKL-40/Aβ42 ratios can predict risk of developing cognitive impairment [9], [15], [137], this minimal panel of six CSF biomarkers (YKL-40, NrCAM, chromogranin A, carnosinase I, tau and Aβ42) begins to segregate individuals into six clinicopathological categories: normal cognition without amyloid plaques, normal cognition with amyloid plaques (preclinical AD), normal cognition at increased risk to develop dementia (converters), very mild dementia (CDR 0.5), very mild dementia at increased risk for progression, and mild dementia (CDR 1) (Figure 7).

We acknowledge that this minimal panel of biomarkers currently has insufficient sensitivity and specificity for clinical application, particularly because it has not been fully evaluated for its ability to discriminate AD from non-AD causes of dementia (although Aβ42, p-tau181, tau, and specific fragments of chromogranin A and cystatin C have shown some ability to distinguish AD from frontotemporal lobar degeneration [FTLD]) [22], [226], [227]. The incorporation of additional biomarkers that are likely to discriminate early AD from cognitive normalcy, such as those identified in the first phase of this study, or other biomarkers that have already shown promise for distinguishing AD from other leading causes of dementia (e.g. agouti related peptide, eotaxin-3, and hepatocyte growth factor [19], complement C3a des-arg and integral membrane protein 2B CT [22], for FTLDs; and alpha-synuclein [228], apoH and vitamin D binding protein [25] for Lewy body disorders), would likely improve the panel's diagnostic utility. However, even in its current form, this initial panel might show value if applied in the context of clinical trial design, wherein simple enrichment of study populations for characteristics of interest would increase efficiency and power and reduce duration and cost. A biomarker panel like this one might also allow clinical trials to evaluate stage-specific responses to treatment, which may differ. Finally, because most of these biomarkers reflect underlying pathological changes in real time, it is appealing to speculate that these biomarkers may have additional utility for evaluating clinically imperceptible treatment responses (as in [229]) and for monitoring neuropathological – rather than cognitive – decline.

## Supporting Information

Figure S1 **ApoE protein isoforms appear in different gel features on 2D-DIGE.** Overlays of fluorescent 2D-DIGE images from gels representing CSF from two individuals with homozygosity for *APOE-ε2* (green) or *APOE-ε3* (red) (panel **A**) and for *APOE-ε3* (green) or *APOE-ε4* (red) (panel **B**) illustrate the heterogeneity of signal distribution by isoelectric point and molecular weight among apoE protein isoforms derived from different alleles. In panels **C**, **D**, **E**, **F**, **G**, **H**, signal intensities of individual CSF samples, grouped by genotype (2/2, 3/3 and 4/4 represent homozygotes; 2/3, 3/4 represent heterozygotes) are indicated for six apoE gel features (labeled C, D, E, F, G, H in panels **A** and **B**), illustrating that gel features C and D represent apoE2; gel feature E represents multiple forms; gel feature F represents apoE3; and gel features G and H, apoE4.
(TIF)

Table S1 **Mass spectrometry and protein identification data for 2D-DIGE gel features that differ in AD CSF.** Results are ordered sequentially by “heat map #” [column A], corresponding to the ‘heat map’ row numbers in Figure 2. “Spot” [column B] refers to BVA number (see Methods). “(Accession) primary protein name” [column C] provides the gi number and protein name from the NCBI database. “Protein molecular weight” [column D] is the gene product molecular weight in Daltons. “Protein score” [column E] is the MASCOT-generated protein score. “Protein ID probability” [column F] indicates Scaffold's percent probability that the protein identification is correct. “Spectral count” [column G] is the number of spectra assigned to the protein by Scaffold. “Unique proteins” [column H] refers to the number of recognized tryptic peptides attributed to the protein by MASCOT. “Peptide sequence” [column I] indicates the amino acid sequence of the tryptic peptide predicted by MASCOT. “MASCOT ion score” [column J] is the MASCOT quality assessment of the peptide sequence assignment. “M/Z (observed)” [column K] is mass/charge ratio. “Mass (observed)” [column L] of peptide is indicated in Daltons. “Mass (theoretical)” [column M] is idealized tryptic peptide mass as predicted by NCBI. “Mass error (ppm)” [column N] is the error in parts per million determined through comparison of theoretical peptide mass to data generated by mass spectrometry. “MS source” [column O] reflects the mass spectrometer that produced the observed data (Q-STAR or LTQ-FT). “Modifications” [column P] lists variable post-translational modifications identified by mass spectrometry peptide sequence analysis.
(XLS)
