# Supplementary figures and images for: Identification and Validation of Novel Cerebrospinal Fluid Biomarkers for Staging Early Alzheimer's Disease
Source: PLoS One. 2011 Jan 12;6(1):e16032. doi: 10.1371/journal.pone.0016032 (PMC3020224; doi:10.1371/journal.pone.0016032)

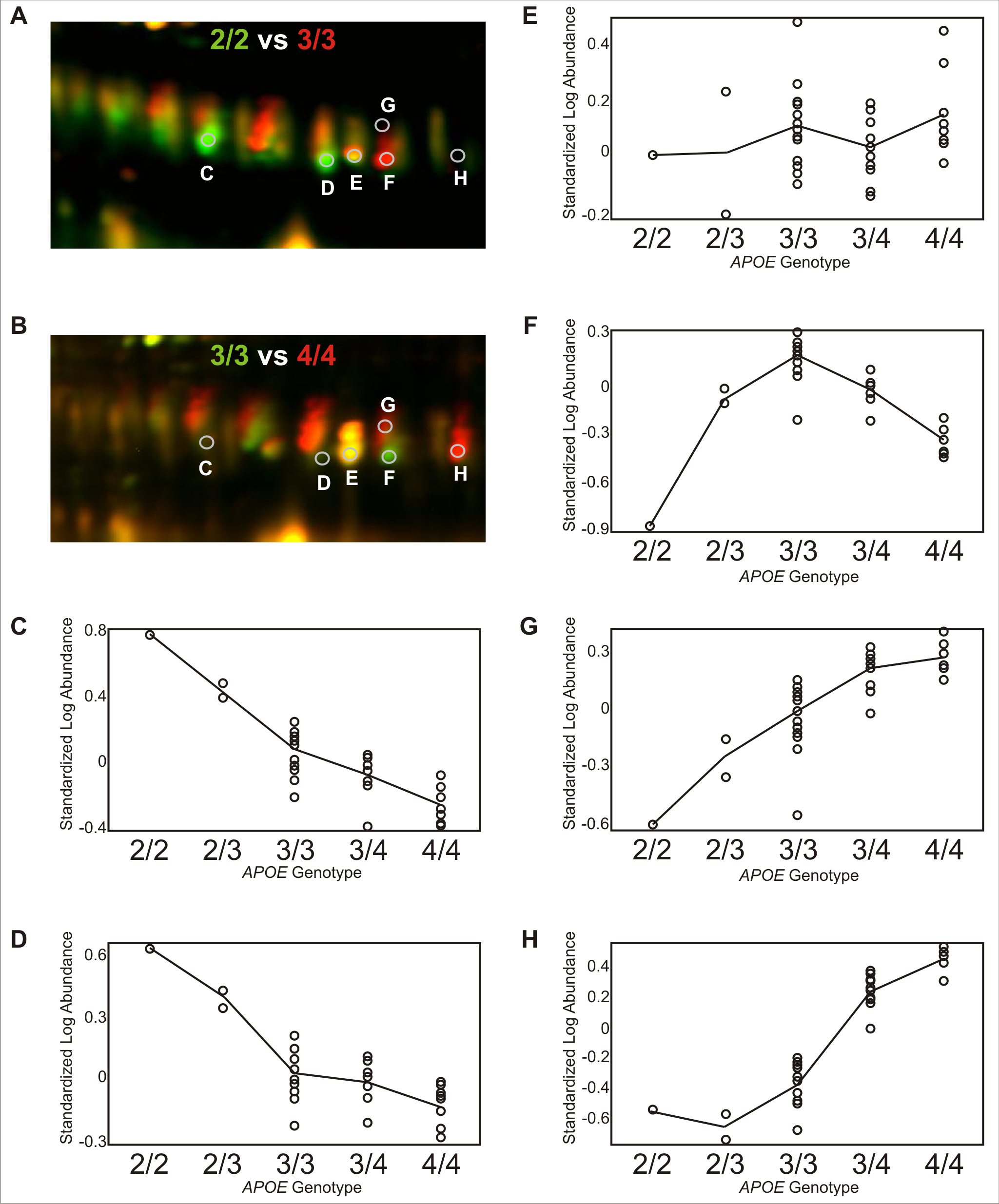

Supplement: Figure S1 — ApoE protein isoforms appear in different gel features on 2D-DIGE. Overlays of fluorescent 2D-DIGE images from gels representing CSF from two individuals with homozygosity for APOE-ε2 (green) or APOE-ε3 (red) (panel A) and for APOE-ε3 (green) or APOE-ε4 (red) (panel B) illustrate the heterogeneity of signal distribution by isoelectric point and molecular weight among apoE protein isoforms derived from different alleles. In panels C, D, E, F, G, H, signal intensities of individual CSF samples, grouped by genotype (2/2, 3/3 and 4/4 represent homozygotes; 2/3, 3/4 represent heterozygotes) are indicated for six apoE gel features (labeled C, D, E, F, G, H in panels A and B), illustrating that gel features C and D represent apoE2; gel feature E represents multiple forms; gel feature F represents apoE3; and gel features G and H, apoE4. (TIF) [file pone.0016032.s001.tif]
